# A SAXS-based approach to rationally evaluate radical scavengers – toward eliminating radiation damage in solution and crystallographic studies

**DOI:** 10.1107/S1600577521004045

**Published:** 2021-08-09

**Authors:** Timothy R. Stachowski, Mary E. Snell, Edward H. Snell

**Affiliations:** a Hauptman-Woodward Medical Research Institute, 700 Ellicott St, Buffalo, NY 14203, USA; b Cell Stress Biology, Roswell Park Comprehensive Cancer Center, 665 Elm Street, Buffalo, NY 14203, USA; c Materials Design and Innovation, State University at New York at Buffalo, 700 Ellicott St, Buffalo, NY 14203, USA

**Keywords:** radiation damage, SAXS, scavengers, protein structure, di­sulfide bond

## Abstract

X-ray-based techniques are a powerful tool in structural biology but the radiation-induced chemistry that results can be detrimental and may mask an accurate structural understanding. In the crystallographic case, cryocooling has been employed as a successful mitigation strategy but also has its limitations including the trapping of non-biological structural states. Crystallographic and solution studies performed at physiological temperatures can reveal otherwise hidden but relevant conformations, but are limited by their increased susceptibility to radiation damage. In this case, chemical additives that scavenge the species generated by radiation can mitigate damage but are not always successful and the mechanisms are often unclear. Using a protein designed to undergo a large-scale structural change from breakage of a di­sulfide bond, radiation damage can be monitored with small-angle X-ray scattering. Using this, we have quantitatively evaluated how three scavengers commonly used in crystallographic experiments – sodium nitrate, cysteine, and ascorbic acid – perform in solution at 10°C. Sodium nitrate was the most effective scavenger and completely inhibited fragmentation of the di­sulfide bond at a lower concentration (500 µ*M*) compared with cysteine (∼5 m*M*) while ascorbic acid performed best at 5 m*M* but could only reduce fragmentation by ∼75% after a total accumulated dose of 792 Gy. The relative effectiveness of each scavenger matches their reported affinities for solvated electrons. Saturating concentrations of each scavenger shifted fragmentation from first order to a zeroth-order process, perhaps indicating the direct contribution of photoabsorption. The SAXS-based method can detect damage at X-ray doses far lower than those accessible crystallographically, thereby providing a detailed picture of scavenger processes. The solution results are also in close agreement with what is known about scavenger performance and mechanism in a crystallographic setting and suggest that a link can be made between the damage phenomenon in the two scenarios. Therefore, our engineered approach might provide a platform for more systematic and comprehensive screening of radioprotectants that can directly inform mitigation strategies for both solution and crystallographic experiments, while also clarifying fundamental radiation damage mechanisms.

## Abstract 2

A system engineered to produce a large-scale structural change from X-ray-induced di­sulfide bond cleavage allows residue-specific changes to be interpreted with SAXS. This system is used to investigate how radical scavengers reduce bond breakage during data collection at 10°C with the results providing insight into mitigation strategies for crystallographic and solution experiments.

## 1. Introduction   

X-ray techniques provide key information on biological mechanisms at the molecular and protein level resolution. However, a limitation of these techniques can be the radiation chemistry involved. X-rays deposit energy inelastically in the sample, damaging it through primary and secondary processes. Primary damage is mainly due to direct photo-absorption of energy leading to an electron ejection from the atom. Secondary damage is from the formation of highly reactive products generated from the ionization of protein atoms and the photolysis of water that then further interact with the sample. These processes have been studied in detail through X-ray crystallographic studies. Global damage manifests in the overall data statistics, and specific damage in structural changes to specific residues in the resultant model, which can misdirect the biologic interpretation. The current state of our knowledge in this area is nicely summarized elsewhere (Garman & Weik, 2019 ▸).

Cryocooling crystals during X-ray data collection is an effective method of reducing radiation damage, preventing the diffusion of most solvent-generated radicals, although it does not completely prevent the damage (Gonzalez & Nave, 1994 ▸; Symons, 1995 ▸). Solvated electrons remain motile even at cryogenic temperatures (Jones *et al.*, 1987 ▸; Garman, 2010 ▸). Despite the advances that cryo-cooling has enabled, the technique can also mask biologically meaningful conformations (Fraser *et al.*, 2009 ▸, 2011 ▸), yield non-native structural artifacts (Frauenfelder *et al.*, 1987 ▸; Juers & Matthews, 2001 ▸), and may not be applicable to every system.

**Figure 1:**
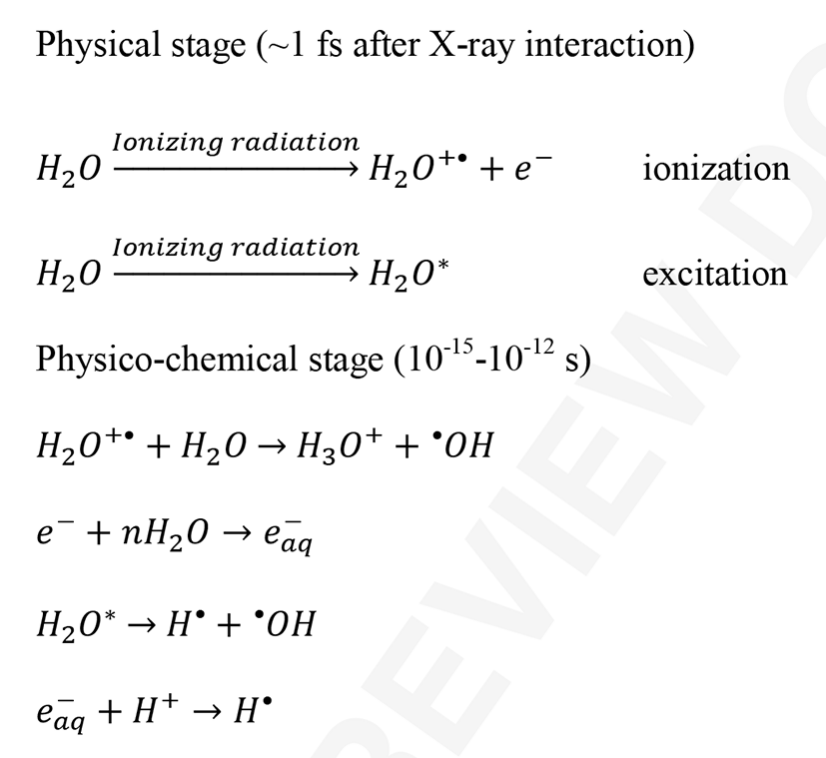
Species produced by secondary damage from the photolysis of water (Ward, 1988 ▸; Southworth-Davies & Garman, 2007 ▸; Le Caër, 2011 ▸). The asterisk signifies a molecule or atom in an excited state.

An approach to mitigating damage at room temperature is to chemically scavenge the radicals that are generated during radiation exposure. The photolysis of water by X-rays generates highly reactive products mainly consisting of solvated electrons (e^−^), hydroxyl radicals (HO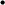), and hydro­nium ions (H_3_O^+^) (Fig. 1 ▸). Scavengers function by intercepting these and other species and converting them into less reactive species with lower mobility before they react with the protein (Barker *et al.*, 2009 ▸), or by repairing incompletely damaged residues (O’Neill *et al.*, 2002 ▸). This reduces the potential for damage to the protein and has been a successful approach in both cryogenic (Murray & Garman, 2002 ▸; Southworth-Davies *et al.*, 2007 ▸) and room-temperature crystallographic studies (Zaloga & Sarma, 1974 ▸; Sarma & Zaloga, 1975 ▸; Cascio *et al.*, 1984 ▸; Barker *et al.*, 2009 ▸).

While scavengers have been used with some success, knowledge that guides their effective use is confusing. Studies suggesting that particular scavengers are both effective and ineffective have been reported based on data collected in practically identical ways (Allan *et al.*, 2013 ▸). The effectiveness of a scavenger for a particular system is not well predicted *a priori* and the protective mechanisms are not well understood, limiting their widespread use. Further complicating the successful use of scavengers is that some also have the potential to affect the structure themselves. For example, by-products from radical–nitrate interactions were observed to cause damage to aromatic residues (Shi *et al.*, 2011 ▸). The lack of detailed knowledge can be attributed in part to the limitations of crystallographic studies of radioprotection such as the spatial and temporal averaging in crystals, the influence of buffer components necessary for crystallization, and the varying metrics used to monitor damage (Allan *et al.*, 2013 ▸).

**Figure 2:**
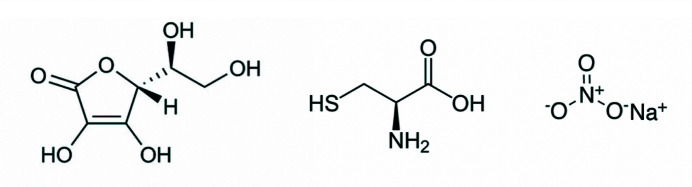
The three scavengers tested in this study: (left) ascorbic acid (ascorbate), (middle) l-cysteine, and (right) sodium nitrate.

The most sensitive residues to primary radiation damage are cysteines and me­thio­nines due to the high photo-absorption cross section and electron-affinity of the sulfur linkage (Ravelli & McSweeney, 2000 ▸). Di­sulfides are important components in many protein mechanisms and damage to these motifs has the potential to misdirect biologic interpretations. Many crystallographic studies have focused on di­sulfide bond damage to understand the mechanism and develop strategies to mitigate the impact of radiation damage overall at cryogenic temperature (Ravelli & McSweeney, 2000 ▸; Sutton *et al.*, 2013 ▸; O’Neill *et al.*, 2002 ▸), room temperature (Gotthard *et al.*, 2019 ▸; Southworth-Davies *et al.*, 2007 ▸) and both (de la Mora *et al.*, 2020 ▸; Russi *et al.*, 2017 ▸). In the specific case of X-ray-induced di­sulfide bond damage, ascorbic acid at room temperature (Barker *et al.*, 2009 ▸), and sodium nitrate at cryogenic temperature (de la Mora *et al.*, 2011 ▸), and cysteine (Kmetko *et al.*, 2011 ▸) at both room and cryogenic temperatures have shown promise (Fig. 2 ▸). At room temperature, ascorbic acid is a strong scavenger of 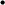
OH, with a *k*_•OH_ = 8.0 × 10^9^ *M* ^−1^ s^−1^ (Buxton *et al.*, 1988 ▸), but weakly intercepts solvated electrons, *k*_e^−^(aq)_ = 3.0 × 10^8^ *M* ^−1^ s^−1^ (Schuler *et al.*, 1974 ▸). Cysteine and cystine have moderate affinity for solvated electrons with *k*_e^−^(aq)_ > 5.0 × 10^9^ *M* ^−1^ s^−1^ (Anbar, 1969 ▸). Reduction of nitrate by solvated electrons is fastest with a rate of *k*_e^−^(aq)_ = 9.7 × 10^9^ *M* ^−1^ s^−1^ (Grätzel *et al.*, 1970 ▸). Ascorbate readily undergoes two consecutive yet reversible one-electron oxidation processes that form an ascorbate radical as an intermediate. Pairs of these radicals react to produce ascorbate and de­hydro­ascorbate (Du *et al.*, 2012 ▸). The reaction of 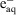 with reduced cysteine generates the hydro­sulfide ion (HS^−^) and the corresponding alkyl radical (*R* 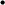) (Reisz *et al.*, 2014 ▸). Sodium nitrate undergoes a one-electron reduction forming NO_3_^2−^ before decomposing to NO_2_ (Grätzel *et al.*, 1970 ▸; de la Mora *et al.*, 2011 ▸). A reaction with hydroxyl yields nitric acid, NHO_3_, that has the potential to regenerate the nitrate ion following deprotonation (Allan *et al.*, 2013 ▸).

Small-angle X-ray scattering (SAXS) is a solution technique that can provide low-resolution structural information. Because it is a solution technique, it is not constrained to studies under the conditions where a crystal is grown and can be preserved. The influence of scavengers on global damage in SAXS has been studied (Brooks-Bartlett *et al.*, 2017 ▸; Kuwamoto *et al.*, 2004 ▸; Crosas *et al.*, 2017 ▸; Castellví *et al.*, 2020 ▸; Jeffries *et al.*, 2015 ▸), but alterations to specific motifs by incident X-rays has until recently not been resolvable due to the limited resolution of the technique. Previously, we reported a sensitive and quantitative tool to evaluate radiation damage to di­sulfide bonds using SAXS from doses less than 100 Gy at 10°C, a fraction of the crystallographic dose (Stachowski *et al.*, 2021 ▸). This tool comprises an engineered mutant of endoglycosidase-H that dimerizes through an accessible and radiation-sensitive di­sulfide bond (Stachowski *et al.*, 2021 ▸). Cleavage of the di­sulfide bond by X-rays results in fragmentation of the dimer into two equally sized monomers and a readily observable signal in the SAXS data. Using this, di­sulfide cleavage can be detected, and the impact of scavengers assessed. Here, we use this radiation damage reporting system to quantitatively assess ascorbic acid, sodium nitrate, and l-cysteine scavengers in protecting di­sulfide bonds from radiation damage.

## 2. Materials and methods   

### 2.1. Sample preparation   

Endoglycosidase-H (endoH) was engineered to dimerize through an interchain di­sulfide bond (endoH_CYS_), and purified as previously described (Stachowski *et al.*, 2021 ▸). The three scavengers studied – ascorbic acid, l-cysteine, and sodium nitrate – were chosen by considering their accessibility, prior use in crystallography, radical affinity (*i.e.* oxidative or reductive), and reported scavenging effects (*i.e.* global or specific) (Allan *et al.*, 2013 ▸). Seven concentrations of each of the three scavengers ranging from 50 n*M* to 50 m*M* were tested to explore the range commonly used in both crystallographic and other radiation chemistry studies. Since fragmentation of the dimer was monitored through relative changes in the integrated intensity, a matching buffer for profile subtraction was not necessary. This allowed for directly adding scavengers to the protein solution, increasing the throughput of the method, and eliminating a significant source of potential error from buffer mismatch. Specifically, the protein was concentrated to 10.0 mg ml^−1^ in 20 m*M* Tris-HCl, pH 7.5, 50 m*M* NaCl, and 1.0 m*M* EDTA before being added directly to an equal volume of the same buffer containing varying concentrations of each scavenger (prepared through serial dilution) so that the final concentration of protein used in the SAXS experiments was 5.0 mg ml^−1^ (95 µ*M*). A sample containing (1) buffer and 50 m*M* scavenger and (2) a sample with protein and buffer but without any scavenger were used as controls. The buffer pH was specifically chosen to ensure that a dimer/monomer transition results from irradiation (Stachowski *et al.*, 2021 ▸). The pH of the buffer and scavengers were measured as they were prepared at 22°C and then at 10°C, the regular temperature of data collection on the beamline used. Tris-HCl is temperature sensitive and there was an expected increase of approximately half a pH unit for the l-cysteine and sodium nitrate solutions on cooling, consistent across concentrations. A similar shift was seen for ascorbic acid except at higher concentrations where the ascorbic acid concentration is outside the buffering capacity and drives a pH change, shifting it for the 5 m*M* and 50 m*M* concentrations to pH 6.66 and 3.46 at 22°C, and to 7.20 and 3.58 at 10°C, respectively (Fig. S1 of the supporting information).

### 2.2. SAXS data collection   

Data were collected at the Advanced Light Source (ALS) SIBYLS beamline using the high-throughput mail-in SAXS service (Dyer *et al.*, 2014 ▸). A volume of 25 µl of each sample held at 10°C was loaded into the sample chamber. A single dose series was collected for each sample where the exposure time for each frame was 0.3 s and a total of 33 frames were collected for each sample in a static position, following a previous protocol (Stachowski *et al.*, 2021 ▸). The photon energy used was 10.2 keV (1.216 Å). Momentum-transfer values were calculated as *q* = 4πsinθ/λ, where 2θ is the scattering angle and λ is the X-ray wavelength in Å. Data were recorded using a PILATUS 2M detector (Dectris; Switzerland). Samples were kept at 10°C during data collection. The data collection parameters are summarized in Table S1.

### 2.3. Absorbed dose calculations   

The unattenuated flux was experimentally determined as 1.13 × 10^12^ photons s^−1^ based on the unimodal density of water at ambient conditions (Clark *et al.*, 2010 ▸). The beam profile details were supplied by the beamline staff. *RADDOSE-3D* modified for SAXS experiments conducted in a cuboidal sample cell (Brooks-Bartlett *et al.*, 2017 ▸) was used for calculating the dose rate (80 Gy s^−1^), taking into account the attenuation by the sample container window (12%), window material (mica), window diameter (20 µm), the beam type (top-hat), and the rectangular beam area (3.4 mm^2^). The cell path length was 1.3 mm ± 0.1 mm. The short exposure time and large beam size are such that diffusion of damaged or undamaged material into and out of the beam path (Hopkins & Thorne, 2016 ▸) does not have a noticeable impact on the dose. The parameters used for calculating dose are available in Table S2.

### 2.4. SAXS data processing   

The integrated intensity of each scattering curve was calculated using a custom Python script (Stachowski *et al.*, 2019 ▸, 2021 ▸) from *q* ≃ 0.01 to *q* ≃ 0.06 Å^−1^, which was the range where the decrease in intensity corresponding to fragmentation of the dimer was most pronounced. Relative changes in the integrated intensity in this region against dose were used as a metric to assess radioprotective ability (Hopkins & Thorne, 2016 ▸). In contrast to global damage (Hopkins & Thorne, 2016 ▸), previously reported single value decomposition and a volume fraction analysis on buffer subtracted profiles show that fragmentation from X-ray exposure is highly reproducible and yields only monomer (cleaved dimer) and dimer species alone (Stachowski *et al.*, 2021 ▸). For this reason, buffer subtraction was not required, and quantitating the effectiveness of scavengers could be achieved by comparing relative changes in integrated intensity. Since only relative changes in integrated intensity were of interest in this study, the data were not placed on an absolute intensity scale before analysis. Based on data from the companion study in Stachowski *et al.* (2021 ▸), the errors in the integrated intensity were estimated at less than 1% across the dose series and at most 2.3% in a single data point (Fig. S2). Fitting of intensity decays to kinetic equations was performed using the LinearModelFit and NonlinearModelFit functions in *Mathematica*. A first-order process was described by the integrated form, *A* = *A*_0_exp(−*kd*) + *C*, where *A* is the integrated intensity at some dose (arbitrary units), *A*_0_ is the integrated intensity at the initial dose (arbitrary units), *k* is the rate constant (Gy^−1^), *d* is the dose (Gy) and *C* is a constant. The half-life for a first-order process was calculated by 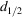 = 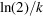. A zeroth-order process was described by the integrated form, *A* = *A*_0_ − *kd* + *C*, where the symbols are defined as for the first-order equation above except for *k* which is in *A*Gy^−1^. The half-life for a zeroth-order process was estimated by *d*_1/2_ = *A*_0_/2*k*. The coefficients obtained from fitting the scattering data to kinetic descriptions are available in Table 1 ▸.

## 3. Results   

### 3.1. Fragmentation can be monitored in the raw scattering profiles   

**Figure 3:**
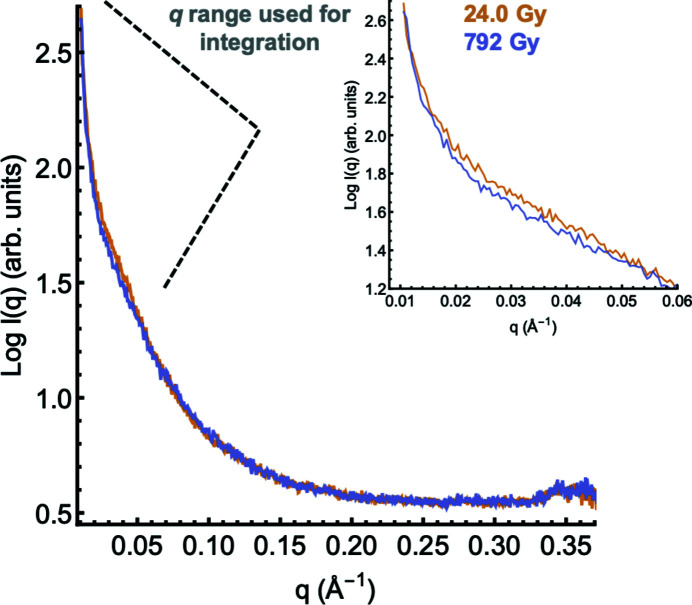
The radially averaged scattering curve of endoH_CYS_ in standard buffer conditions exhibits a decrease in the scattering in the range *q* ≃ 0.01–0.06 between the first dose (24 Gy; blue) and the total accumulated dose (792 Gy; gold) indicating fragmentation. The inset shows the scattering data only in the range used for integration.

In the protein control without a scavenger, the production of the cleaved monomers was apparent in the scattering profiles by an X-ray dose-dependent decrease in intensity in the low- and mid-*q* regions (*q* ≃ 0.01 to *q* ≃ 0.06 Å^−1^) (Fig. 3 ▸). This occurred from X-ray doses significantly less than those used to monitor damage to di­sulfides in crystallographic studies. The change in the relative integrated intensity of the scattering curve in this region with respect to the absorbed dose (Gy, J kg^−1^) provided a baseline to evaluate scavenging ability, given that cleavage of some sample will have occurred within this first exposure.

### 3.2. The effectiveness of scavengers is strongly concentration-dependent   

**Figure 4:**
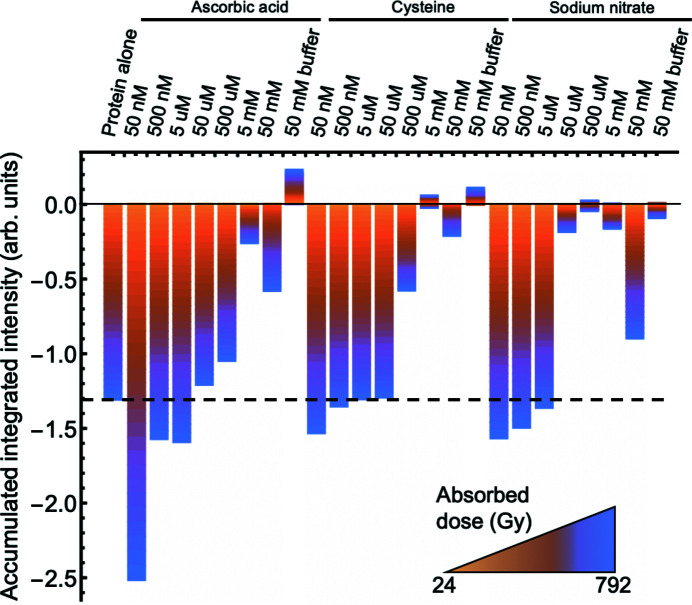
Scavengers exhibit concentration-dependent changes in integrated intensity. The effectiveness of each scavenger (ascorbic acid, cysteine, and sodium nitrate) to inhibit fragmentation was judged by comparing relative X-ray dose-driven changes in the accumulated integrated intensity (AII) from 24 Gy (orange) to 792 Gy (purple). This method provides the running total integrated intensity as the total X-ray dose increases. Column heights that show the smallest deviation from zero indicate the most effective damage protection. Each scavenger was tested at seven concentrations ranging from 50 n*M* to 50 m*M* in addition to the highest concentration of scavenger in the absence of protein (50 m*M* buffer). Each scavenger exhibited concentration-dependent effects. Low concentrations (50 n*M*–5 µ*M*) increased the amount of fragmentation while higher concentrations (500 µ*M*–5 m*M*) mitigated the damage. The effectiveness of each scavenger at a particular concentration also differed. The horizontal dashed line refers to the final AII value (1.35) of the control after 792 Gy.

The effectiveness of each scavenger in inhibiting fragmentation was first evaluated from the relative X-ray dose-driven changes in accumulated integrated intensity (AII) (Fig. 4 ▸). This metric provides the running total integrated intensity as X-ray dose accumulates. AII was calculated by normalizing the integrated intensity of each scattering curve to that of the first dose point (24 Gy) for each respective dose series. From there a value of 1.0 was subtracted from every data point so that a more negative AII indicates more fragmentation (damage) and matches the direction of change in the intensity of the raw scattering patterns (Fig. 3 ▸). An AII of zero indicates that there is no measurable fragmentation. After the total accumulated dose of 792 Gy, low concentrations of each scavenger (50 n*M*–50 µ*M*) exhibited more fragmentation than the protein irradiated without a scavenger (control). This effect was most notable in samples containing low concentrations of ascorbic acid. For example, the total AII of 50 n*M* ascorbic acid was about twofold lower than the control. Higher concentrations (50 µ*M*–5 m*M*) of each scavenger all yielded a total AII greater than the control, suggesting that each scavenger was able to mitigate some damage. The relative effectiveness of each scavenger differed at a particular concentration. As showed by a high AII, sodium nitrate greatly reduced fragmentation at 50 µ*M* and was completely effective at 500 µ*M* where AII remained near zero across all doses. Cysteine was less effective with protection beginning at 500 µ*M* and complete elimination of measurable damage seen at 5 m*M*. Ascorbic acid was seen to offer protection to the bond starting at 50 µ*M* and was most effective at 5 m*M* where it exhibited an AII ∼25% greater than that of the control. The 50 m*M* concentrations of each scavenger were less effective than their 5 m*M* counterpart. Buffer controls of 50 m*M* cysteine or ascorbic acid without protein also exhibited total AIIs greater than zero. This might indicate that the reduced mitigations observed at 50 m*M* are underestimated. Without correction, these changes in buffer intensity could cause buffer subtraction errors if full processing of the data with unsubtracted scattering profiles was desired. This necessitates careful control for buffer subtraction errors if the SAXS profiles are to be used for structural studies. In summary, each of the scavengers tested in this study was successful in inhibiting X-ray-induced di­sulfide bond cleavage in solution, but the effectiveness of each scavenger at a particular concentration differed, suggesting distinct chemical protective mechanisms.

**Figure 5:**
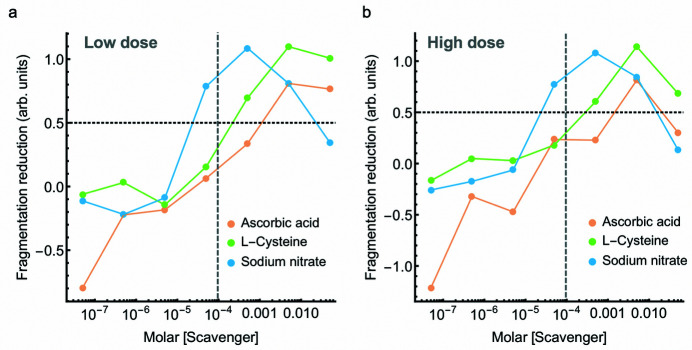
The relative effectiveness of each scavenger is largely consistent across X-ray doses. (*a*) Scavenger titrations at a low dose point (120 Gy) and (*b*) at the total accumulated dose (792 Gy). Integrated intensities were compared for seven concentrations for ascorbic acid (orange), cysteine (green), and sodium nitrate (blue). Fragmentation reduction (FR) was calculated from the difference between the integrated intensity of the scavenger and the control which was then normalized to the integrated intensity of the control and subtracted from 1.0. In this way, at a particular dose point, an FR of zero represents an amount of fragmentation equal to that of the control and an FR of 1.0 is equal to complete mitigation of fragmentation. The horizontal dashed line represents a fragmentation reduction of 50%. The gray vertical dashed line represents the concentration of protein (95 µ*M*).

Irradiating the engineered protein in the presence of scavengers changed the total amount of fragmentation (Fig. 4 ▸). To determine if the effectiveness of each scavenger is consistent at different X-ray doses, the amount of fragmentation was compared between two dose points. Based on a volume fraction analysis reported in our previous study, most fragmentation occurred with accumulated doses less than ∼250 Gy (3.0 s) and was negligible beyond ∼600 Gy (6.0 s) (Stachowski *et al.*, 2021 ▸). Building on these results and by looking at relative changes in intensity in this study (Fig. 5 ▸), 120 Gy was chosen as the ‘low’ dose that contained an appreciable amount of fragmented dimer but the reaction had not proceeded to completion. 792 Gy was chosen as the ‘high’ X-ray dose containing the maximal amount of fragmented dimer. A metric called fragmentation reduction (FR) was developed and describes the protection provided by the scavenger. This is calculated from the difference between the integrated intensity of the scavenger and the control which is then normalized to the control integrated intensity alone (protein with no scavenger). By subtracting this from unity, an FR of zero represents an amount of fragmentation equal to that of the control and an FR of 1.0 is equal to complete mitigation of fragmentation – effective elimination of di­sulfide radiation damage. Comparing this metric between low and high dose points indicates that the relative effectiveness of each scavenger at a particular concentration is largely consistent across the X-ray doses tested. The one exception is the 50 m*M* concentration of each scavenger, the highest concentration tested, which becomes progressively less effective as the X-ray dose increases. Importantly, for significant mitigation of fragmentation, scavengers were needed at concentrations near or greater than the protein concentration. Interpolating between measured data points shows that, to reduce fragmentation by 50% after 792 Gy, ascorbic acid (1 m*M*) and cysteine (400 µ*M*) required concentrations approximately 10.5 and 4.2 times greater than the protein concentration (95 µ*M*), respectively. Sodium nitrate was more effective and reduced more than 50% of fragmentation at 30 µ*M* (3.06 times less the protein concentration) and eliminated fragmentation at about 500 µ*M* (5.3 times greater than the protein concentration) after 792 Gy. The concentration of sodium nitrate needed for complete mitigation (500 µ*M*) was about an order of magnitude less than required for cysteine (∼5 m*M*). The effectiveness of each scavenger is concentration-dependent and the relative effectiveness of each scavenger at a particular concentration differs.

### 3.3. Scavengers alter the kinetics of fragmentation   

**Figure 6:**
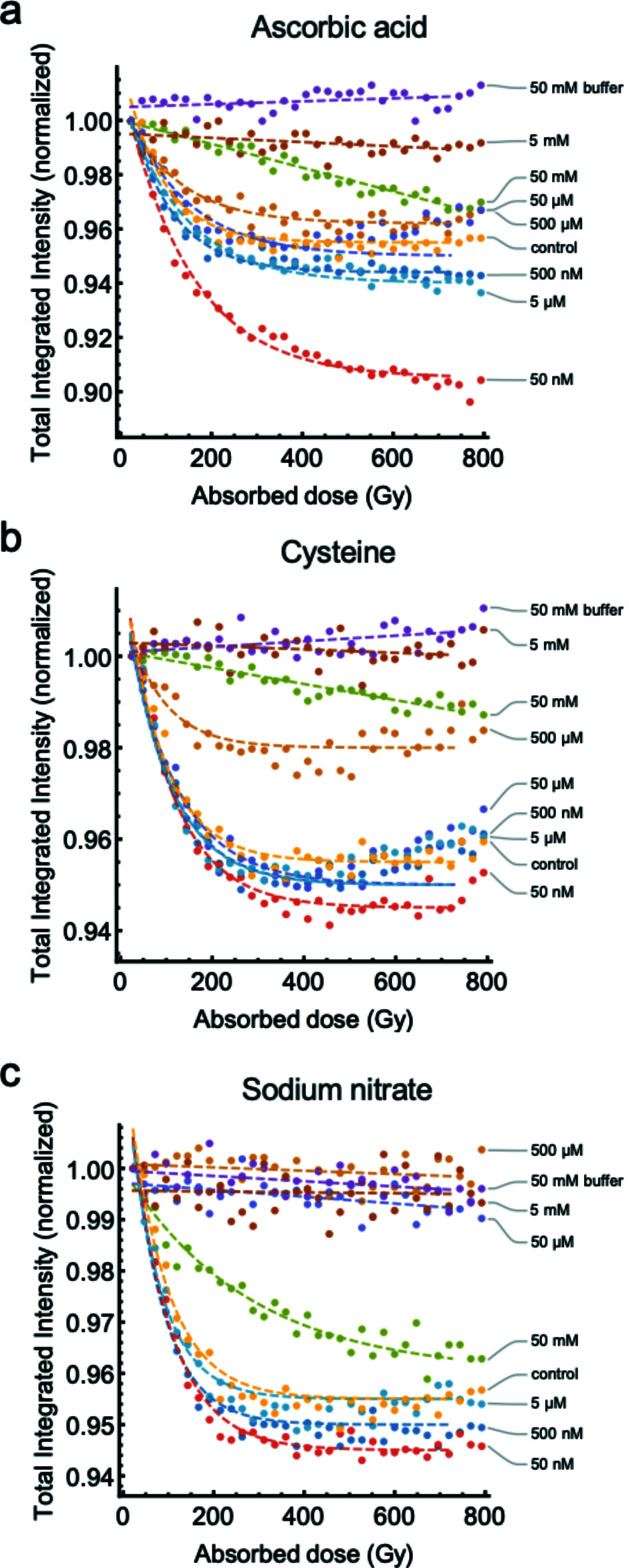
Scavengers alter the kinetics of fragmentation. Three scavengers (*a*) ascorbic acid, (*b*) cysteine, and (*c*) sodium nitrate were tested for their abilities to mitigate fragmentation of the engineered di­sulfide bond. Each scavenger was tested at seven concentrations: 50 n*M* (red), 500 n*M* (dark blue), 5 µ*M* (light blue), 50 µ*M* (light purple), 500 µ*M* (orange), 5 m*M* (brown), 50 m*M* (green) and compared with the X-ray dose-dependent integrated intensity trajectory of the control (no scavenger; yellow) and the 50 m*M* buffer (dark purple) of each respective scavenger. (Dashed lines) Scavengers at low concentrations exhibit exponential decays in integrated intensity that reflect a first-order process whereas at high concentrations the integrated intensity decay becomes linear reflecting a zeroth-order process.

**Figure 7:**
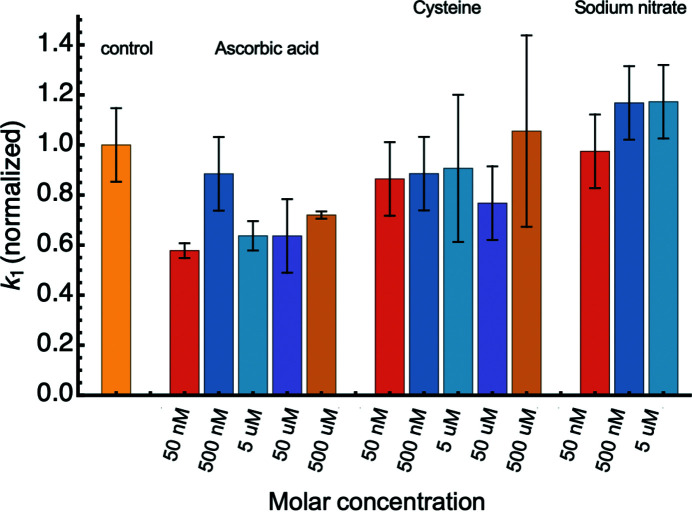
Reaction rates at sub-inhibiting concentrations suggest scavenging mechanisms. Integrated intensity trajectories at low concentrations that could be described by a first-order process were normalized to the reaction rate of the control. Error bars represent the standard error from fitting to an exponential decay.

Fragmentation of the engineered protein in the absence of a scavenger exhibits an exponential decay that is characteristic of a first-order process (Stachowski *et al.*, 2021 ▸). Fitting the integrated intensity trajectories of the scavenger titrations (Fig. 6 ▸) to kinetic descriptions yields the parameters described in Table 1 ▸. Low concentrations of ascorbic acid scavenger (50 n*M*–5 µ*M*) showed more damage in the protein than the protein without scavengers (Fig. 4 ▸). At a slightly higher scavenger concentration, 50 µ*M*, there was a modest reduction in the total amount of fragmentation with the protection being maximum at 5 m*M*. The fragmentations indicated by the integrated intensity decay of the buffer control and the 50 n*M*–500 µ*M* ascorbic acid concentration data are well described by a first-order exponential equation [Fig. 6 ▸(*a*)] that have slightly reduced reaction rates as concentration increases (Table 1 ▸ and Fig. 7 ▸). The integrated intensity decay at low concentrations stabilized once the accumulated dose reached 400 Gy with the exception of 50 n*M* ascorbic acid, indicating that the reactions have reached completion and were not furthered by additional X-ray dose. In contrast to low concentrations, the 5 m*M* and 50 m*M* ascorbic acid scavenger concentrations exhibited linear integrated intensity decays, reflecting a zeroth-order process with half-lives several orders of magnitude larger than the control (Table 1 ▸). This phenomenon was more pronounced at 50 m*M* than at 5 m*M* ascorbic acid where the pH was inconsistent with the other concentrations (Fig. S1). The observed difference between a linear and exponential decay in these data are unambiguous, similar to Barker *et al.* (2009 ▸).

Low concentrations of cysteine (50 n*M*–5 µ*M*) yielded more fragmentation compared with the control but less than corresponding concentrations of ascorbic acid (Fig. 4 ▸). Importantly, these concentrations of cysteine all showed a slight dose-dependent increase in integrated intensity following 400 Gy, where the control reaction reached completion (Fig. 6 ▸). This increase in integrated intensity following an initial decrease was not systematically apparent in the results from other scavengers or the control, and if only the endpoint of the reaction is monitored it might give the false impression that cysteine is more effective [Fig. 5 ▸(*b*)]. Reaction rates at these concentrations were less than the control but higher than the same concentrations of ascorbic acid (Table 1 ▸, Fig. 7 ▸). At higher concentrations, 500 µ*M* cysteine exhibited a modest reduction in the intensity decay but remained a first-order process with a slightly greater reaction rate compared with the control. The 5 m*M* and 50 m*M* concentrations both exhibited linear integrated intensity decays that agree with a zero-order description similar to the ascorbic acid case.

For sodium nitrate, complete prevention of measurable damage occurred at concentrations between 50 µ*M* and 5 m*M* making it the most effective scavenger of the three tested (Fig. 5 ▸). Low concentrations (50 n*M* and 500 n*M*) of sodium nitrate slightly increased damage of the protein (Fig. 4 ▸) and showed increased reaction rates compared with the control (Table 1 ▸) and with the other scavengers on average (Fig. 7 ▸). 50 m*M* sodium nitrate performed worse than 5 m*M*, which was also the case with ascorbic acid and cysteine. Notably, unlike the other two scavengers that were best described as zeroth-order processes, the performance of 50 m*M* sodium nitrate remained a first-order process.

## 4. Discussion   

Using the engineered protein to examine the effects of different scavenger on an exposed di­sulfide bond produced quantitative data allowing the scavenger performance to be compared in a sensitive and rapid manner. Although these results are only for a limited number of scavengers, the analysis can be easily extended to evaluate any other scavenger. Chemical changes may occur to the scavenger but had a minimal impact on the buffer blank in this study, allowing the direct scattering curves to be evaluated. Perhaps the most important finding of this work is that the effectiveness of a particular scavenger might be related to how it alters the rate of fragmentation. Scavengers that had faster reaction rates at low (sub-inhibiting) concentrations were more effective and completely inhibited fragmentation at lower concentrations. Scavengers with lower reaction rates also increased the total amount of fragmentation at low concentrations compared with corresponding concentrations of scavengers with faster reaction rates. Specifically, the total amount of fragmentation in the presence of sub-inhibiting concentrations of sodium nitrate increased compared with the control but was less than corresponding concentrations of ascorbic acid, where the reaction rate was lower (Figs. 4 ▸ and 7 ▸).

Global damage (aggregation) in SAXS has been extensively studied, albeit at much higher X-ray doses, and, while it can have a delayed onset (Brooks-Bartlett *et al.*, 2017 ▸; Castellví *et al.*, 2020 ▸; Kuwamoto *et al.*, 2004 ▸), it is strongly dependent on X-ray dose (Crosas *et al.*, 2017 ▸; Hopkins & Thorne, 2016 ▸; Kuwamoto *et al.*, 2004 ▸) indicating a zeroth-order reaction. On the other hand, the intensity decay that corresponds to site-specific fragmentation of the protein alone (Stachowski *et al.*, 2021 ▸) and with low concentrations of scavengers can be described as a first-order reaction making it distinct from global damage. This description of the intensity trajectory suggests that the reaction is triggered by X-rays but is perhaps more dependent on the concentration of undamaged protein than the X-ray dose. In the absence of a scavenger or with low scavenger concentrations, fragmentation could be described as a first-order reaction (Stachowski *et al.*, 2021 ▸). Additional fragmentation was limited beyond 600 Gy despite the incomplete conversion of the dimer to monomer. This plateau effect is distinct from global damage (aggregation) in SAXS, which is strongly dependent on X-ray dose (Crosas *et al.*, 2017 ▸; Hopkins & Thorne, 2016 ▸; Kuwamoto *et al.*, 2004 ▸) and indicates that the system reaches an equilibrium despite additional X-ray doses. This is supported by a volume fraction analysis that monitored the proportion of dimer and monomer populations across X-ray dose and saw no significant change past 600 Gy (Stachowski *et al.*, 2021 ▸).

The plateau effect and the behavior of the scavengers are most likely due to a combination of factors including (1) the rate of diffusion or radicals through the solvent, and (2) quenching and conversion of radicals. These factors have also been identified in damage that occurs in room-temperature crystallographic experiments (Owen *et al.*, 2012 ▸). With the data produced by our SAXS studies, we can explore the mechanism behind the scavenging effect. Free radicals are not generated uniformly in the solvent but form along regions called spurs (Hill & Smith, 1994 ▸). For every 100 eV of energy absorbed under anaerobic conditions, 4.14 H_2_O, 2.7 H^+^, 2.7 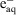, and 2.87 
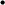
OH number of molecules are formed (Buxton, 1987 ▸). To cause damage to a di­sulfide bond, a sufficient number of 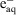 must travel a certain distance in a finite amount of time to encounter the bond. Estimations place the lifetime of solvated electrons in the microsecond range and the 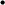
OH in the nanosecond range, which reflects the greater reactivity of the 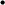
OH (Roots & Okada, 1975 ▸). Similarly, the solvated electron can diffuse thousands of angstroms while the 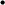
OH is limited to local interactions of less than 100 Å (Roots & Okada, 1975 ▸).

Because many biological buffers already contain strong 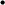
OH scavengers (Allan *et al.*, 2013 ▸), including Tris and sodium chloride used in the sample buffer for this study, avoiding damage to di­sulfide bonds mainly focuses on scavenging electrons. Each scavenger tested here can intercept 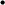
OH and solvated electrons but with different efficiencies. As previously noted, ascorbic acid is a strong scavenger of 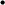
OH, *k*_•OH_ = 8.0 × 10^9^ *M* ^−1^ s^−1^ (Buxton *et al.*, 1988 ▸), but weakly intercepts solvated electrons, *k*_e^−^(aq)_ = 3.0 × 10^8^ *M* ^−1^ s^−1^ (Schuler *et al.*, 1974 ▸). Cysteine and cystine have moderate affinity for solvated electrons with *k*_e^−^(aq)_ > 5.0 × 10^9^ *M* ^−1^ s^−1^ (Anbar, 1969 ▸). Reduction of nitrate by solvated electrons is fastest with a rate of *k*_e^−^(aq)_ = 9.7 × 10^9^ *M* ^−1^ s^−1^ (Grätzel *et al.*, 1970 ▸). These relative rates of electron conversion are in agreement with both the relative efficacy of each scavenger (Fig. 5 ▸) and the relative fragmentation rates (Table 1 ▸, Fig. 7 ▸). Based on the relative rates of solvated electron conversion and the concentration of sodium nitrate that completely inhibited fragmentation (500 µ*M*; Figs. 4 ▸ and 6 ▸), equivalent inhibition for the other scavengers can be predicted to occur at approximately 16 m*M* ascorbic acid and 1.0 m*M* for cysteine, which are in reasonable agreement with the values measured here (Fig. 5 ▸). Ascorbic acid, which is preferential to 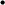
OH, performed best at 5.0 m*M* but was only able to inhibit ∼75% of fragmentation. Similar to the crystallographic mechanism (Sutton *et al.*, 2013 ▸), this is evidence that practically 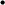
OH is not important for mitigating di­sulfide bond cleavage in solution (assuming the experiments are performed with a typical biological buffer). These results suggest that the plateau effect might reflect an underlying equilibrium between the generation and quenching of radicals that is below the threshold to drive further fragmentation (Stachowski *et al.*, 2021 ▸). On the other hand, scavenging occurs through the conversion of highly reactive species to less reactive but still reactive ones. As stated previously, buffer components, such as the buffering agent or salt, which are known to scavenger hydroxyl radicals (Buxton *et al.*, 1988 ▸) and were present in each sample tested here, could play a critical role in converting radicals to ones that contribute to cleavage (Simpson *et al.*, 1988 ▸). These species may not be regenerated during the experiment and might also contribute to the plateau effect.

Degassing has been tested as a potential method to reduce radiation damage, minimizing the dissolved oxygen (Hopkins & Thorne, 2016 ▸). Slightly faster damage was seen in that study for degassed solutions, however large standard deviations precluded definite conclusions. Degassing is not a routine approach with SAXS, but the beamline used has a positive pressure helium environment (to minimize air scatter and for oxygen-sensitive samples) so degassed fluids could be tested while maintained in their degassed state (Hura *et al.*, 2009 ▸). Sheath flow has been used to reduce radiation damage in a flowing system by increasing sample velocity through the beam (Kirby *et al.*, 2016 ▸). The laminar flow minimizes sample dwell on the chamber walls, but can be disrupted by bubble formation. Degassing is used to minimize the release of absorbed gases in the X-ray beam, and enables faster sample flow but is not used directly to alter the radiation chemistry taking place. The impact of dissolved oxygen on damage is not clear, with observations of decreasing and increasing damage (Saha *et al.*, 1995 ▸). Degassing was not used here and the potential impact of degassing remains an open question.

Low concentrations of scavengers promoted fragmentation (Fig. 4 ▸). Why this is the case is unclear. This phenomenon was most profound in ascorbic acid, which is known to react with free metals, particularly iron, to generate radicals through Fenton chemistry (Stadtman & Berlett, 1991 ▸). Although heavy metals were not a deliberate component in the buffer, these metals may be present in small amounts and could react with low concentrations of ascorbic acid. Increased damage was not observed at higher concentrations of ascorbic acid, suggesting that excess ascorbic acid concentrations can scavenge these radicals in the same way as those generated from X-ray exposure. Scavenging these two processes simultaneously could explain the overall limited effectiveness of ascorbic acid compared with the other scavengers.

Each scavenger exhibited maximum efficiency at a particular concentration. Exceeding these concentrations was often detrimental and yielded more fragmentation (Figs. 4 ▸ and 6 ▸). As for the effects at low concentrations, why this is the case is also unclear, but this is not the first observation of the phenomenon. Brooks-Bartlett *et al.* (2017 ▸) tested the ability of several scavengers to reduce global damage in SAXS experiments, including ascorbic acid and sodium nitrate. The authors note that, while many compounds exhibited strong concentration-dependent reductions in damage, some components exhibited a weak or negative correlation at high concentrations, such as DTT. This was not observed for ascorbic acid or sodium nitrate, but these compounds were only tested up to 10 m*M*, whereas in the present study this phenomenon becomes noticeable for ascorbic acid at 5 m*M* and beyond where pH becomes an important consideration given the buffering capacity. The reactivity of electrons with cysteine is pH-dependent, with reactivity increasing as pH is reduced (Poole, 2015 ▸). This may have an impact in the higher concentration ascorbic acid scavenger studies. However, the results from these studies underscore the idea that complete compensation when using a less effective scavenger cannot be achieved by blindly using higher concentrations.

At saturating scavenger concentrations, the fragmentation indicated by the decay in the integrated intensity shifts from exponential to linear, which is characteristic of a zeroth-order process. The weak linear decay might illustrate the effects of direct damage to the protein, *i.e.* direct photo-absorption of the di­sulfide bond, in the absence of damage from radicals in the solvent. A similar relationship between scavenging and intensity decay has been noted in room-temperature crystallographic scavenger studies on ascorbate and 1,4-benzo­quinone (Barker *et al.*, 2009 ▸). In our study, each scavenger tested exhibited a linear decay in integrated intensity at 5 m*M* while cysteine and ascorbic acid had a more profound decay at 50 m*M* (Fig. 6 ▸, Table 1 ▸). The different response between the two concentrations is unlikely to be due to scavengers influencing photo-absorption of the di­sulfide bonds but perhaps reflects the persisting influence of solution chemistries such as ionic strength or pH on the dissociation of the dimer.

While room-temperature crystallographic damage studies are often performed with X-ray doses much higher than SAXS experiments and have not explored the concentration dependence of scavengers as extensively as this study, some connection can be made between results from the two techniques. The physical processes involved in both cases are the same, and crystals have a relatively high solvent content of 27–78% (Matthews, 1968 ▸, 1976 ▸). Experimental studies support this connection. Tetragonal chicken egg-while lysozyme (commonly referred to as HEWL) crystals, which are based on typical solvent content and contain approximately 59 m*M* HEWL and 236 m*M* concentration of di­sulfide bonds (4 per monomer), are a common model system for radiation damage studies (Murray & Garman, 2002 ▸). Barker *et al.* (2009 ▸) report positive mitigation of site-specific damage to HEWL at room temperature using 500 m*M* ascorbic acid, about twice the di­sulfide bond concentration. Here, all three scavengers did not show appreciable mitigation of damage until they reached a concentration near or greater than the concentration of the protein (95 µ*M* protein, 43 µ*M* di­sulfide). The higher ratio of ascorbic acid to protein di­sulfide required to completely inhibit cleavage in the solution state compared with the crystal (Barker *et al.*, 2009 ▸) is not surprising considering the increased susceptibility of the engineered bond to damaging species in solution compared with being restricted to channels within the crystal lattice. Results from both techniques agree that the amount of scavenger should greatly exceed the protein concentration to achieve complete mitigation and the discrepancy noted here might reflect a possible conversion factor to relate information between the two techniques. On the other hand, 500 m*M* sodium nitrate was able to quench spectrophotometric detection of electrons during room-temperature collection from HEWL crystals (Allan *et al.*, 2013 ▸). This is much greater than the estimate from SAXS, but it is unknown if lower concentrations would perform similarly as they were not investigated in the HEWL study. Cysteine is a known radiosensitizer in solution (Shimazu & Tappel, 1964 ▸) but has not exhibited strong efficacy in crystallographic studies (Allan *et al.*, 2013 ▸). For example, 100–200 m*M* cysteine exhibited either no protection or exacerbated global damage to lysozyme crystals at room temperature (Kmetko *et al.*, 2011 ▸). The deleterious effect of cysteine is also apparent in these SAXS experiments where the initial fragmentation occurred with a similar trajectory to the control, but was followed by an increase in integrated intensity at doses exceeding 400 Gy (Fig. 6 ▸). This did not occur systematically in the other scavengers or the control. The increase in integrated intensity is most likely to be due to reactivity between radicalized free cysteine in solution and the protein leading to protein crosslinking, and has been hypothesized by other groups to explain the poor performance of cysteine as a scavenger in crystallographic experiments (Kmetko *et al.*, 2011 ▸).

Historically there has always been interest in room or near physiological temperature data collection. This interest has grown in light of developments that reduce radiation damage such as serial crystallographic methods (de la Mora *et al.*, 2020 ▸), X-ray free-electron lasers (Nass, 2019 ▸), and low dose collection strategies (Stellato *et al.*, 2014 ▸). However, these approaches, while promising, can be experimentally challenging. They can require sample delivery systems, high-frame-rate detectors, and algorithms that filter, assemble, and scale the data from the large number of diffraction patterns of the many individual crystals necessary to create complete datasets (Stellato *et al.*, 2014 ▸). There has also been an approach to introduce cryocooling techniques to SAXS (Meisburger *et al.*, 2013 ▸) but this has not had the same impact on the field as cryocooling in X-ray crystallography. Scavengers represent an experimentally simple approach to reducing secondary damage at room temperature. However, the results concerning the effectiveness of scavengers have been contradictory or unclear (Allan *et al.*, 2013 ▸). Both X-ray crystallographic and SAXS studies suffer from primary damage, and scavengers do not prevent this, which may impact the interpretation of results. Similarly, although there is experimental evidence that scavengers perform a similar role in both solution and the crystallographic state, it is not clear if the impact is similar. Brooks-Bartlett *et al.* (2017 ▸) discuss this in detail with a *D*_Thresh_ value, the threshold dose for evidence of damage to be seen in the data, that differs for room-temperature SAXS experiments and other types of X-ray diffraction experiment at both room and cryogenic temperatures. Some of these observations depend on the exact definition of *D*_Thresh_. We have developed a sensitive and quantitative tool to evaluate radiation damage to di­sulfide bonds using SAXS that might overcome these limitations and provide a clearer understanding of how scavengers mitigate damage and potentially improve their effectiveness in structural studies, both in solution and in the crystallographic case.

Another important biomedical application of radiation is for cancer treatment where doses of 2.0–30 Gy are used for moderate and high-dose radiotherapy (Vaiserman *et al.*, 2018 ▸; Timmerman *et al.*, 2010 ▸; Gao *et al.*, 2019 ▸; Lo *et al.*, 2010 ▸). *In vivo* and *in vitro* experiments indicate that certain proteins contain motifs that render them selectively more sensitive to radiation-induced oxidation or reduction, and more so than for other proteins (Daly, 2012 ▸; Reisz *et al.*, 2014 ▸). Studies show that this sensitivity is sometimes functional as it allows proteins to sense radiation-induced changes in the solution chemistry. Often these proteins are positioned so that they can communicate these environmental changes by initiating and coordinating the stress response pathways that ultimately orchestrate the biological response to radiation (Guéguen *et al.*, 2019 ▸). Our SAXS studies on engineered systems and those of therapeutic importance have used doses down to 36.3 Gy (Stachowski *et al.*, 2021 ▸) and 14.2 Gy (Stachowski *et al.*, 2019 ▸), respectively, to study how low doses of X-rays influence protein structure. However, these types of studies are challenging, as the signal necessary to collect quality data exceeds the low doses that trigger these structural processes in a biologically relevant way. Through an understanding of scavenger mechanism and effectiveness, one might be able to introduce scavengers to biological systems so that the perceived biological dose is reduced to a therapeutic level without sacrificing sufficient signal for structural studies. This could allow us to explore the potential structural impact that therapeutic treatments might cause.

While the scavengers themselves may influence the observations of biological mechanism, the remarkably low concentrations that produce a noticeable effect suggest that the di­sulfide cleavage pathway is one that can be pursued with a quantitative understanding of their mechanism. However, a note of caution should be sounded. The studies presented here focus on one damage mechanism, that of free-radical attack on the di­sulfide bond. The success of this scavenging is likely to be a clear indicator of processes that protect other residues against attack, but we have not demonstrated that in this study. There may also be protein-specific effects, but our engineered protein approach represents a deliberately worst-case scenario.

Moreover, it is likely that the scavengers studied here have chemical properties, distinct from their primary scavenging role, that influence di­sulfide bond cleavage. While the separate contribution of each property of a molecule cannot be disentangled here, the strength of this method is that the total effect can be quantitatively measured. The ability of the scavenger to reduce fragmentation while not altering the biological system is what is practically important. Similarly, the results reported here suggest a relationship between protein concentration and scavenger concentration where a scavenger must be at a higher concentration than the protein to be maximally effective. However, it is not clear if this relationship holds at varying protein concentrations. The efficacy of a scavenger is also related to the abundance of radicals in solution which is in turn dependent on factors related to the X-ray beam parameters such as flux. Ultimately, this approach can robustly monitor this damage process and potentially identify molecules with radioprotective properties and also experimental factors for further analysis.

The results presented here are promising, showing that there is an agreement between X-ray induced di­sulfide bond cleavage derived crystallographically and phenomena occurring in solution. Monitoring relative changes in integrated intensity between three scavengers showed relationships that mirrored the relative affinities for solvated electrons. The approach used opens the door for more comprehensive and extensive screening of chemicals or a combination of chemicals to understand how they influence radiation damage mechanism and clarify mechanisms of radiation chemistry involved with solution studies and both room temperature and cryo-crystallography. The approach presents a key element towards an actionable mechanistic understanding of scavenger use to reduce and potentially eliminate radiation damage in solution and crystallographic studies.

## Supplementary Material

Zip file of scattering curves in text format for all SAXS data. DOI: 10.1107/S1600577521004045/gm5077sup1.zip

Supporting Figures S1 and S2; Tables S1 and S2. DOI: 10.1107/S1600577521004045/gm5077sup2.pdf

## Figures and Tables

**Table 1:** Coefficients of fits for exponential and linear functions to the data from the sample without any scavenger (control) and with the three scavengers tested (ascorbic acid, cysteine, sodium nitrate)

	Exponential (first-order)†			Linear (zeroth order)†			Half-life
Sample	*A* = *A*_0_exp(−*kd*) + *C*			*A* = *A*_0_ − *kd* + *C*			*d*_1/2_ (Gy)
Control							
	*A*_0_ = 1.023 ± 0.004	*k*_1_ = 0.012 ± 0.001	*C* = 0.955				59.0

Ascorbic acid							
50 n*M*	*A*_0_ = 1.0149 ± 0.0031	*k*_1_ = 0.0068 ± 0.0002	*C* = 0.905				102
500 n*M*	*A*_0_ = 1.016 ± 0.003	*k*_1_ = 0.010 ± 0.001	*C* = 0.944				66.7
5 µ*M*	*A*_0_ = 1.0970 ± 0.0032	*k*_1_ = 0.0075 ± 0.0004	*C* = 0.940				92.6
50 µ*M*	*A*_0_ = 1.010 ± 0.008	*k*_1_ = 0.007 ± 0.001	*C* = 0.950				92.6
500 µ*M*	*A*_0_ = 1.007 ± 0.003	*k*_1_ = 0.008 ± 0.001	*C* = 0.962				81.9
5 m*M*				*A*_0_ = 0.995 ± 0.001	*k*_0_ = 7.785 × 10^−6^ ± 2.70 × 10^−6^	*C* = 0.0	6.39 × 10^4^
50 m*M*				*A*_0_ = 1.000 ± 0.001	*k*_0_ = 4.370 × 10^−5^ ± 1.761 × 10^−6^	*C* = 0.0	1.14 × 10^4^
50 m*M* buffer				*A*_0_ = 1.005 ± 0.001	*k*_0_ = −5.392 × 10^−6^ ± 2.622 × 10^−6^	*C* = 0.0	−9.32 × 10^4^

Cysteine							
50 n*M*	*A*_0_ = 1.024 ± 0.004	*k*_1_ = 0.010 ± 0.001	*C* = 0.945				68.2
500 n*M*	*A*_0_ = 1.018 ± 0.006	*k*_1_ = 0.010 ± 0.001	*C* = 0.950				66.6
5 µ*M*	*A*_0_ = 1.020 ± 0.008	*k*_1_ = 0.011 ± 0.002	*C* = 0.950				65.1
50 µ*M*	*A*_0_ = 1.015 ± 0.007	*k*_1_ = 0.009 ± 0.001	*C* = 0.950				76.9
500 µ*M*	*A*_0_ = 1.013 ± 0.006	*k*_1_ = 0.012 ± 0.003	*C* = 0.980				55.9
5 m*M*				*A*_0_ = 1.003 ± 0.001	*k*_0_ = 3.751 × 10^−6^ ± 2.245 × 10^−6^	*C* = 0.0	1.34 × 10^5^
50 m*M*				*A*_0_ = 1.001 ± 0.001	*k*_0_ = 1.767 × 10^−5^ ± 1.147 × 10^−6^	*C* = 0.0	2.83 × 10^4^
50 m*M* buffer				*A*_0_ = 1.000 ± 0.001	*k*_0_ = −6.151 × 10^−6^ ± 1.96 × 10^−6^	*C* = 0.0	−8.14 × 10^5^
					
Sodium nitrate							
50 n*M*	*A*_0_ = 1.023 ± 0.003	*k*_1_ = 0.011 ± 0.001	*C* = 0.945				60.5
500 n*M*	*A*_0_ = 1.026 ± 0.004	*k*_1_ = 0.0137 ± 0.001	*C* = 0.950				50.5
5 µ*M*	*A*_0_ = 1.022 ± 0.005	*k*_1_ = 0.014 ± 0.001	*C* = 0.955				25.1
50 µ*M*				*A*_0_ = 0997 ± 0.001	*k*_1_ = 6.671 × 10^−6^ ± 1.886 × 10^−6^	*C* = 0.0	7.47 × 10^4^
500 µ*M*				*A*_0_ = 1.001 ± 0.001	*k*_0_ = 3.279 × 10^−6^ ± 2.074 × 10^−6^	*C* = 0.0	1.52 × 10^5^
5 m*M*				*A*_0_ = 0.996 ± 0.001	*k*_0_ = 8.796 × 10^−7^ ± 3.204 × 10^−6^	*C* = 0.0	5.652 × 10^5^
50 m*M*	*A*_0_ = 1.000 ± 0.002	*k*_1_ = 0.0036 ± 0.0002	*C* = 0.960				96.0
50 m*M* buffer				*A*_0_ = 1.000 ± 0.0020	*k*_0_ = 5.125 × 10^−6^ ± 1.958 × 10^−6^	*C* = 0.0	9.75 × 10^4^

† *k*_1_ is in units of Gy^−1^ while *k*_0_ is in units of *A*Gy^−1^ where *A* is the integrated intensity.
