# Supplementary material for: A SAXS-based approach to rationally evaluate radical scavengers – toward eliminating radiation damage in solution and crystallographic studies
Source: J Synchrotron Radiat. 2021 Aug 9;28(Pt 5):1309–20. doi: 10.1107/S1600577521004045 (PMC8415334; doi:10.1107/S1600577521004045)
Supplement: Supplementary file 2 [file s-28-01309-sup2.pdf]

JOURNAL OF SYNCHROTRON RADIATION

Volume 28 (2021)

Supporting information for article:

A SAXS based approach to rationally evaluate radical scavengers – toward eliminating radiation damage in solution and crystallographic studies

Timothy R. Stachowski, Mary E. Snell and Edward H. Snell

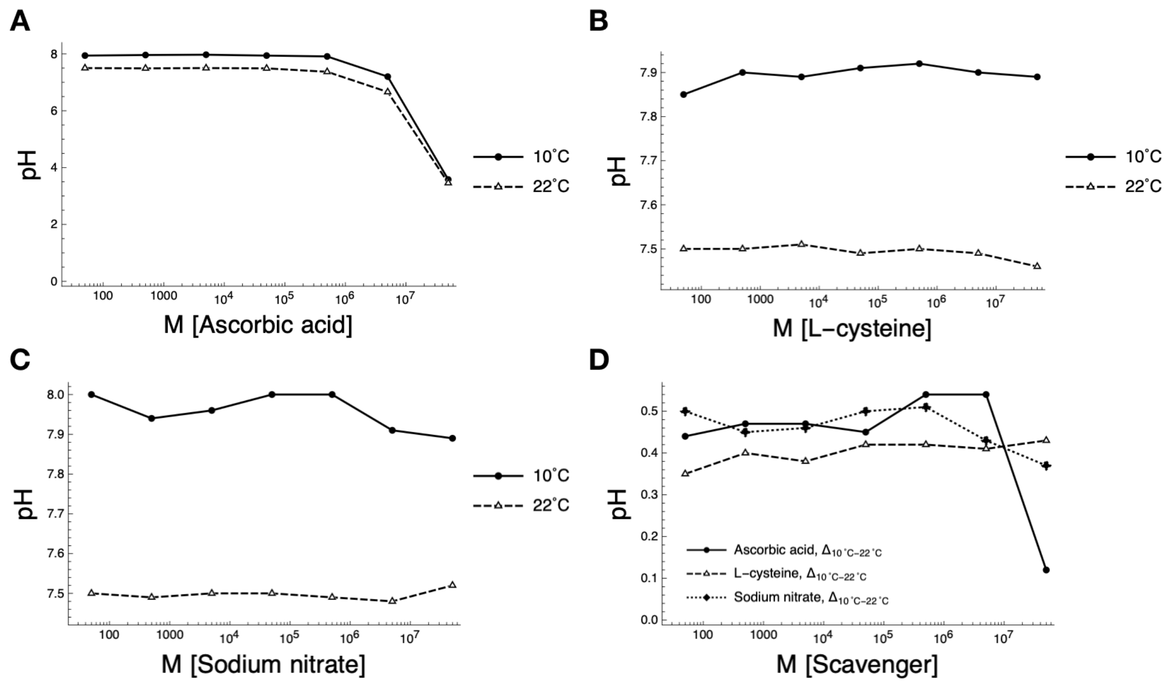

Figure S1 pH values of buffers used with increasing concentrations of (a) ascorbic acid, (b) L-cysteine, and (c) sodium nitrate. pH measurements were taken at 10˚C (solid line) and 22˚C (dotted line). (d) The difference between measurements taken at 10˚C and 22˚C for each concentration of a particular scavenger.

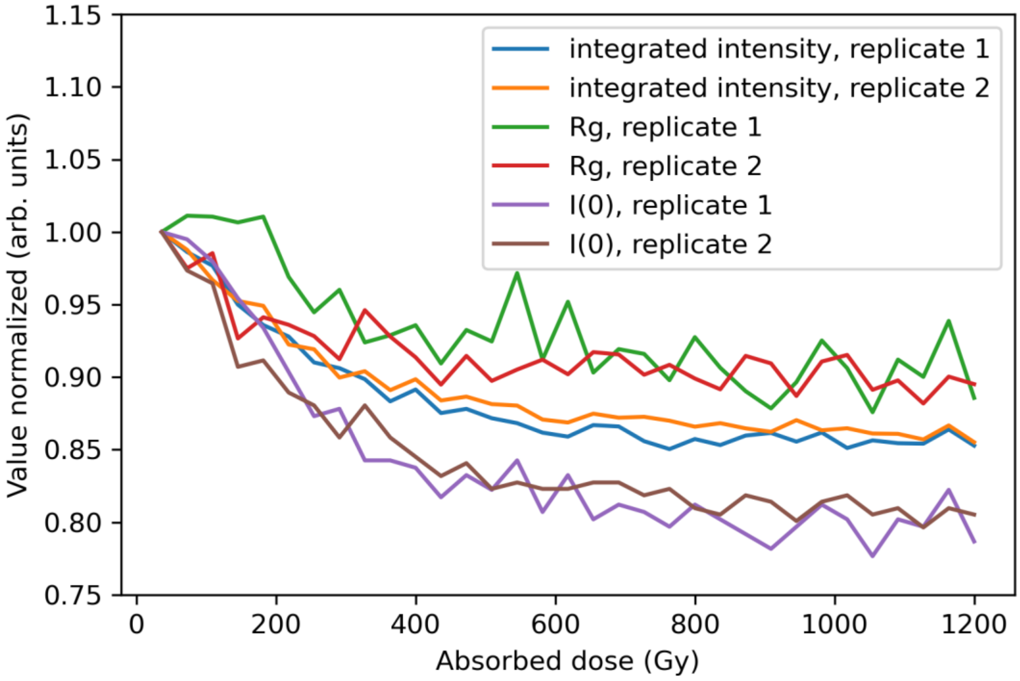

Figure S2 . In the Stachowski et al. (2020) study, two replicates were collected at pH 7.5 and 5.0 mg/ml, close to the conditions reported in this study. The error in integrated intensity is estimated at less than 1% across the dose series and follows a similar dose-dependent decay as I(0) and Rg. Relative changes in integrated intensity provide a proxy for following the fragmentation process.

Table S1 SAXS data collection parameters

Instrument	SIBYLS SAXS Beamline with 2M Detector (Dyer *et al.*, 2014)
Wavelength (Å)	1.216
Camera length (m)	1.4 m
q measurement ($Å^{-1}$)	0.01-0.374
Sample configuration	Static
Temperature	10°C

Table S2 Parameters for X-ray diffraction weighted dose calculations for SAXS experiments using RADDOSE-3D

Unattenuated flux (ph/s)	$1.13x10^{12}$
Sample container window (µm)	20
Sample container material	mica
Attenuation by sample container (%)	12
Beam type	Top-hat
Beam area (mm^2)	3.4
Energy (keV)	10.2
